# Concomitant parathyroid hormone-secreting oncocytic adrenocortical carcinoma and parathyroid adenoma

**DOI:** 10.1210/jcemcr/luag253

**Published:** 2026-09-16

**Authors:** Natalie A Moreno, Amir H Hamrahian, Christian F Meyer, Ciera Mangone, Ezra Baraban, Lilah F Morris-Wiseman

**Affiliations:** Division of Endocrine Surgery, Department of Surgery, Johns Hopkins University School of Medicine, Baltimore, MD 21287, USA; Division of Endocrinology, Department of Medicine, Johns Hopkins University School of Medicine, Baltimore, MD 21287, USA; Division of Oncology, Department of Medicine, Johns Hopkins University School of Medicine, Baltimore, MD 21287, USA; Division of Surgical Pathology, Department of Pathology, Johns Hopkins University School of Medicine, Baltimore, MD 21287, USA; Division of Surgical Pathology, Department of Pathology, Johns Hopkins University School of Medicine, Baltimore, MD 21287, USA; Division of Endocrine Surgery, Department of Surgery, Johns Hopkins University School of Medicine, Baltimore, MD 21287, USA

**Keywords:** hyperparathyroidism, adrenal cortical carcinoma, parathyroid, hypercalcemia, ectopic PTH secretion, oncocytic adrenal tumor

## Abstract

A 53-year-old man underwent resection of a nonfunctional 9 cm oncocytic adrenal cortical carcinoma (ACC) with focal high-grade features. Two years later he presented again, with gynecomastia and elevated levels of estradiol, 11-deoxycortisol, and serum calcium (10.5 mg/dL, SI: 2.62 mmol/L) (reference range 8.4-10.2 mg/dL [SI: 2.10-2.55 mmol/L]). Imaging demonstrated locally recurrent ACC, which was resected. Steroid excess resolved and he started mitotane. Months after, his calcium was 11.9 mg/dL (SI: 2.97 mmol/L), parathyroid hormone (PTH) 209 pg/mL (SI: 22.0 pmol/L) (reference range 15-65 pg/mL [SI: 1.6-6.9 pmol/L]), and parathyroid hormone–related peptide (PTHrp) was undetectable. Germline genetic testing (*TP53, MSH2, MSH6, MLH1, PMS2,* and *MEN1*) was negative; localizing imaging demonstrated possible right inferior parathyroid adenoma. He underwent 4-gland parathyroid exploration. Right inferior parathyroid (460 mg) was excised, superior parathyroids appeared normal, and left inferior parathyroid was not identified. Intraoperative parathyroid hormone (IOPTH) did not drop appropriately. Follow-up calcium rose to 13.1 mg/dL (SI: 3.27 mmol/L) and PTH to 336 pg/mL (SI: 35.6 pmol/L). Repeat imaging demonstrated ACC recurrence. After cytoreductive surgery (nephrectomy and omentectomy) and heated intraperitoneal chemotherapy (HIPEC), calcium and PTH normalized. Staining of the recurrent ACC demonstrated PTH positivity. This is a novel case of PTH-secreting ACC and concomitant parathyroid adenoma.

## Introduction

Adrenal cortical carcinoma (ACC) is a rare and aggressive endocrine cancer with a reported annual incidence of up to 0.5 to 2 cases per million people [1]. It is functional in up to 60% of cases [2], most commonly secreting excess cortisol. Rarely, ACC secretes hormones that are not native to the adrenal gland, with one prior case report of an oncocytic ACC producing both estradiol and parathyroid hormone (PTH) [3].

Primary hyperparathyroidism is most often caused by a single parathyroid adenoma, and less commonly it presents with multigland disease. Primary hyperparathyroidism is rarely associated with ACC in context of hereditary mutations in *MEN1* causing multiple endocrine neoplasia type 1 (MEN1). Persistent hyperparathyroidism following parathyroidectomy (PTX) is most commonly caused by a missed adenoma. We report a unique case of simultaneous PTH-producing recurrent oncocytic ACC and concurrent parathyroid adenoma.

## Case presentation

A 52-year-old man presented with an incidentally discovered left adrenal mass during workup for chest pain. Cross-sectional imaging demonstrated a 6.3-cm left adrenal mass with indeterminate imaging characteristics, including an unenhanced attenuation of 29 Hounsfield units and absolute washout of 29.4%. Biochemical workup, including plasma metanephrines, 24-hour urine free cortisol (26 μg/day [SI: 71.7 nmol/day]; reference range, 5-64 μg/day [SI: 13.8-176.6 nmol/day]) and serum calcium (9.6 mg/dL [SI: 2.39 mmol/L]; reference range, 8.4-10.2 mg/dL [SI: 2.10-2.55 mmol/L]) were within normal limits. The patient underwent elective left laparoscopic adrenalectomy converted to open due to bleeding. Pathology confirmed a 9-cm predominantly low grade oncocytic ACC (<5 mitoses/high powered field [HPF]) with a focal high-grade component (Ki67 up to 20% in an area of lymphovascular invasion with up to 35 mitoses/HPF). Tumor extended to the inked specimen surface. The tumor had 2 major criteria on the Lin-Weiss-Bisceglia scale (high mitotic rate and lymphovascular invasion) and a Helsinki score of 7, classified as American Joint Committee on Cancer (AJCC) Stage II (T2NxM0). Adjuvant mitotane was offered and deferred. Germline genetic testing for hereditary ACC, including *TP53, MSH2, MSH6, MLH1, PMS2,* and *MEN1* mutations, was negative.

**Figure 1:**
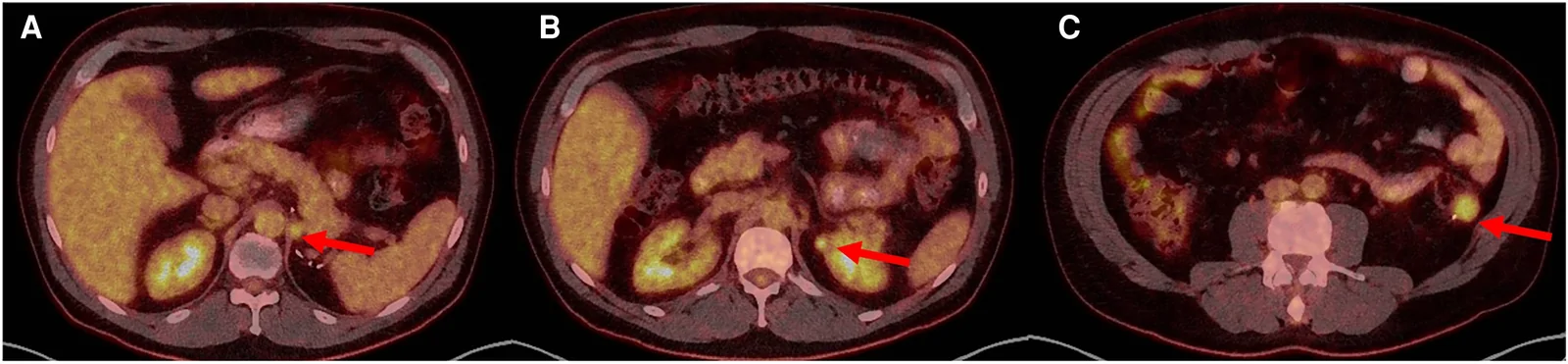
^18^F-fluorodeoxyglucose positron emission tomography (FDG-PET) demonstrating 3 recurrent adrenocortical carcinoma lesions (red arrows) in the left retroperitoneum, including (A) a nodule within the prior suprarenal surgical bed, (B) a nodule adjacent to the left kidney, and (C) a mass inseparable from the descending colon within the left paracolic gutter.

During follow up at 2 years post-resection, ^18^F-fluorodeoxyglucose positron emission tomography (FDG-PET) identified 3 areas of likely tumor recurrence in the left retroperitoneum (Fig. 1). Clinically, the patient had developed gynecomastia and a left lower extremity deep vein thrombosis; he was started on anticoagulation with apixaban. Biochemical workup revealed normal morning cortisol, 17-hydroxyprogesterone, and 17-hydroxypregnenolone, with elevated 11-deoxycortisol, estradiol, dehydroepiandrosterone (DHEA)-sulfate, and calcium (Table 1). He underwent distal pancreatectomy with splenectomy, left partial nephrectomy, and a left partial colectomy, with pathology confirming ACC in 3 separate masses.

**Table 1:** longitudinal laboratory evaluation across the patient's disease course

Laboratory parameter	Reference range	Initial presentation	First ACC recurrence	< 1 month postop first recurrence resection	4-months postop first recurrence resection	Pre-parathyroidectomy	Post-parathyroidectomy	Post-ACC cytoreduction and HIPEC	1 year follow-up
**Calcium-parathyroid axis**									
Calcium	8.4-10.2 mg/dL (2.10-2.55 mmol/L)	9.6 mg/dL (2.40 mmol/L)	**10.5 mg/dL (2.62 mmol/L)**		**12.7 mg/dL (3.17 mmol/L)**	**13.1 mg/dL (3.27 mmol/L)**	**13.5 mg/dL (3.37 mmol/L)**	8.5 mg/dL (2.12 mmol/L)	8.6 mg/dL (2.15 mmol/L)
Ionized calcium	4.53-5.29 mg/dL (1.13-1.32 mmol/L)				**6.45 mg/dL (1.61 mmol/L)**				
PTH	15-65 pg/mL (1.6-6.9 pmol/L)				**138 pg/mL (14.5 pmol/L)**	**209 pg/mL (22 pmol/L)**	**336 pg/mL (35.4 pmol/L)**	20 pg/mL (2.1 pmol/L)	45 pg/mL (4.7 pmol/L)
Phosphorus	2.5-4.5 mg/dL (0.81-1.45 mmol/L)				3 mg/dL (0.97 mmol/L)		**1.7 mg/dL (0.55 mmol/L)**		3.4 mg/dL (1.10 mmol/L)
PTHrp	11-20 pg/mL (1.2-2.1 pmol/L)				< 8 pg/mL (<0.90 pmol/L)				
25-OH vitamin D	30-100 ng/mL (74.9-249.6 nmol/L)				**28 ng/mL (69.9 nmol/L)**				**26.7 ng/mL (66.6 nmol/L)**
**Adrenal hormones**									
Morning cortisol	4.6-23.4 μg/dL (126.9-645.5 nmol/L)	**26** μg/dL **(717.3 nmol/L)**	13.2 μg/dL (364.2 nmol/L)						9.7 μg/dL (267.6 nmol/L)
17-hydroxyprogesterone	37-129 ng/dL (1.12-3.91 nmol/L)		43 ng/dL (1.30 nmol/L)						
17-hydroxypregnenolone	≤ 700 ng/dL (≤ 21.2 nmol/L)		125 ng/dL (3.78 nmol/L)						
11-deoxycortisol	≤ 42 ng/dL (≤ 1.21 nmol/L)		**188 ng/dL (5.42 nmol/L)**	<20 ng/dL (<0.58 nmol/L)					
Estradiol	11.3-43.2 pg/mL (41.5-158.6 pmol/L)		**135 pg/mL (495.6 pmol/L)**	39.9 pg/mL (146.5 pmol/L)	**71 pg/mL (260.6 pmol/L)**				**93.1 pg/mL (341.8 pmol/L)**
DHEA-S	38-313 μg/dL (67.2-112.3 μmol/L)		**336 μg/dL (9.12 μmol/L)**	89 μg/dL (2.42 μmol/L)	98 μg/dL (2.66 μmol/L)				
**Renal/bone markers**									
Creatinine	0.76-1.27 mg/dL (67.2-112.3 μmol/L)		1.26 mg/dL (111.4 μmol/L)						1.23 (108.7 μmol/L)
eGFR	>59 mL/min/1.73 m^2^		80 mL/min/1.73 m^2^						
Alkaline phosphatase	44-121 IU/L		**169 IU/L**						59 IU/L

Abnormal values are shown in bold font. Values in parenthesis are International System of Units (SI).

Abbreviations: 25-OH vitamin D, 25-hydroxy-vitamin D; ACC, adrenocortical carcinoma; DHEA-S, dehydroepiandrosterone sulfate; eGFR, estimated glomerular filtration rate.; HIPEC, cytoreductive surgery plus heated intraperitoneal chemotherapy; PTH, parathyroid hormone; PTHrp, parathyroid hormone–related protein.

Postoperatively, his biochemical markers normalized, and he was initiated on mitotane. Four months later, during routine surveillance, he was found to have a serum calcium level of 12.7 mg/dL (SI: 3.17 mmol/L) and ionized calcium (iCal) of 6.45 mg/dL (SI: 1.61 mmol/L) (reference range 4.53-5.29 mg/dL [SI: 1.13-1.32 mmol/L]).

## Diagnostic assessment

Workup for hypercalcemia was initiated. The patient was taking hydrocortisone with mitotane therapy and there was no biochemical or clinical evidence of adrenal insufficiency. He had no evidence of bony metastases and parathyroid hormone–related peptide (PTHrp) was <8 pg/mL (SI: <0.9 pmol/L) (reference range, 11-20 pg/mL [SI: <2.0 pmol/L]). Workup for primary hyperparathyroidism revealed elevated PTH at 138 pg/mL (SI: 14.5 pmol/L), with mild elevation in alkaline phosphatase 169 IU/L (reference range 44-121 IU/L), and slightly low 25-hydroxy-vitamin D 28 ng/mL (SI: 69.9 nmol/L) (reference range, 30.0-100.0 ng/mL [SI: 74.9-249.6 nmol/L]), while glomerular filtration rate (GFR), creatinine, and phosphorus were within normal limits (Table 1). The presumptive diagnosis was concomitant primary hyperparathyroidism with indications for PTX (calcium >1 mg/dL [SI: >0.25 mmol/L] above normal). Ultrasound showed no parathyroid candidates or thyroid nodules and a 4-dimensional computed tomography (4D-CT) of the neck showed a possible right inferior parathyroid adenoma. Due to the profound hypercalcemia, surgery was planned without further assessment of bone mineral density. He had no history of fractures clinically and no history of nephrolithiasis or nephrocalcinosis detected on imaging.

**Figure 2:**
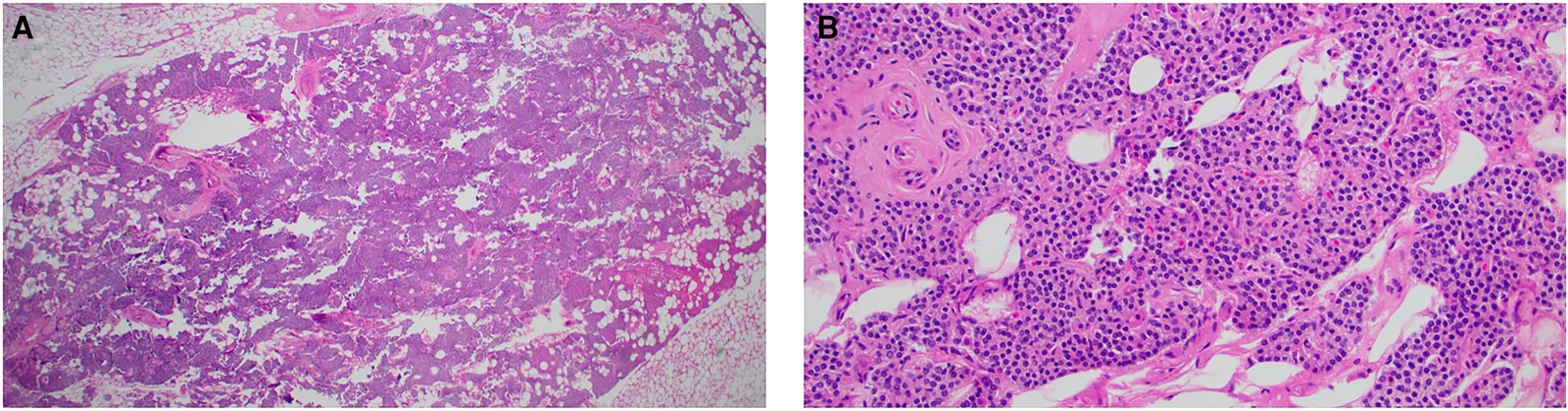
Histologic features of the right inferior parathyroid adenoma. Low power hematoxylin and eosin staining (A) demonstrates proliferation of parathyroid tissue at 2× magnification. Higher power view at 10× magnification demonstrates nests of uniform chief cells and moderate eosinophilic cytoplasm without cytologic atypia (B).

The patient then underwent a 4-gland parathyroid exploration during which an enlarged right inferior parathyroid adenoma weighing 460 mg was resected (Fig. 2). Intraoperatively, this gland appeared to be a benign, enlarged parathyroid gland easily separable from the surrounding tissues without grossly concerning features to suggest a parathyroid malignancy (firmness, discoloration, or hypopigmentation). Due to a lack of clinical suspicion of parathyroid carcinoma, parafibromin immunostaining was not performed. The left superior parathyroid gland appeared enlarged and resected with final pathology revealing a 209-mg piece of tissue with only 20% (42 mg) parathyroid consistent with a normal parathyroid gland. The right superior parathyroid appeared normal, and this was confirmed with biopsy. After a full exploration of the left central neck and thymus, a fourth parathyroid gland was not found. The intraoperative PTH did not drop adequately (pre-excision highest value 198 pg/mL [SI: 20.9 pmol/L] to 30-minute post excision 123 pg/mL [SI: 13.0 pmol/L] as the lowest value in the operating room) and remained elevated postoperatively.

Two weeks following PTX, the calcium rose to 13.5 mg/dL (SI: 3.37 mmol/L) and PTH was 336 pg/mL (SI: 35.4 pmol/L), with phosphorus 1.7 mg/dL (SI: 0.55 mmol/L) (reference range, 2.5-4.5 mg/dL [SI: 0.81-1.45 mmol/L]). The patient was started on cinacalcet. Additional parathyroid imaging including a technetium (Tc)99-sestamibi of the neck and chest was negative. Two months after PTX, interval abdominal imaging for ACC surveillance demonstrated nodularity in the surgical bed concerning for recurrence, confirmed on biopsy.

## Treatment

The patient underwent exploratory laparotomy and cytoreductive surgery with left nephrectomy, omentectomy, and heated intraperitoneal chemotherapy (HIPEC) with cisplatin under clinical trial. Postoperative laboratory data showed near-immediate resolution of hyperparathyroidism and hypercalcemia, with PTH 20 pg/mL (SI: 20 ng/L) and Ca 8.5 mg/dL (SI: 2.12 mmol/L).

## Outcome and follow-up

**Figure 3:**
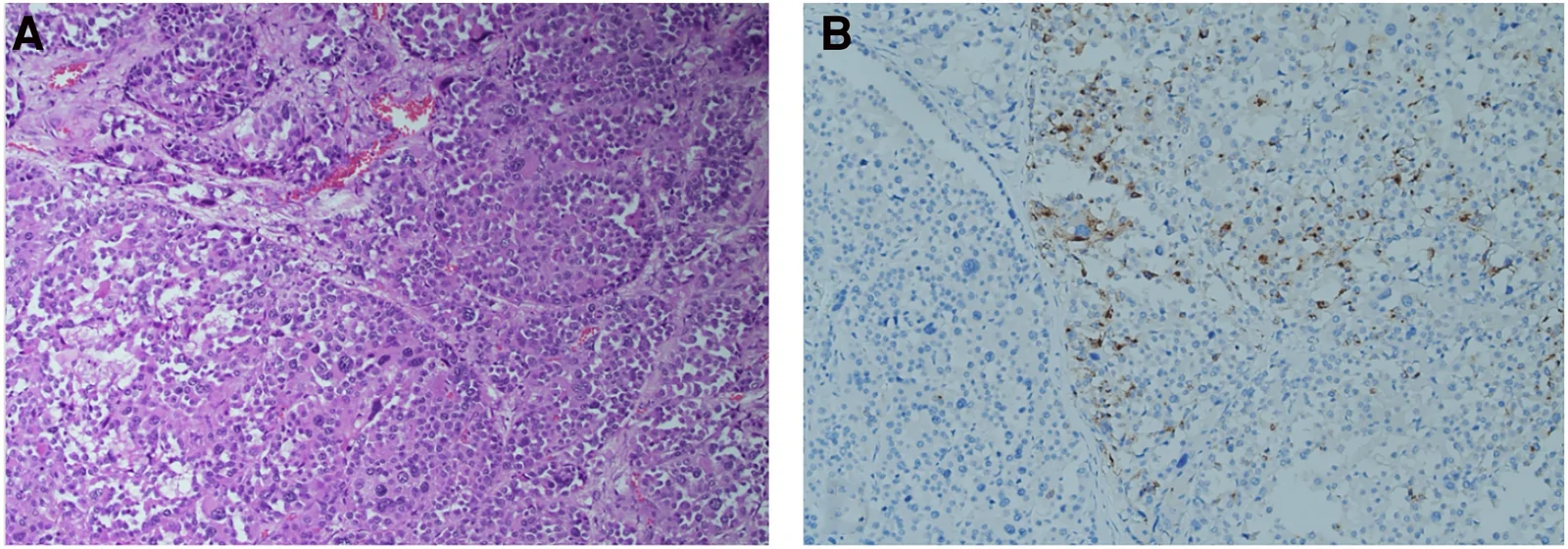
Histologic and immunohistochemical confirmation of ectopic parathyroid hormone (PTH)-producing recurrent adrenocortical carcinoma. Panel A demonstrates hematoxylin and eosin staining showing nests of oncocytic adrenal cortical cells with eosinophilic cytoplasm and trabecular architecture at 10× magnification. Panel B demonstrates patchy positivity within tumor cells confirming ectopic PTH production at 10× magnification.

The patient was discharged home on oral calcitriol, calcium, and hydrocortisone. Pathology confirmed ACC of 3.9 cm involving perinephric tissue and metastatic carcinoma in 3 of 4 lymph nodes and in 2 of 6 para-aortic lymph nodes. One year postoperatively, he has no structural or biochemical evidence of ACC or hyperparathyroidism recurrence. His last calcium level was 8.6 mg/dL (SI: 2.15 mmol/L) with PTH 45 pg/mL (SI: 4.7 pmol/L). He has continued on mitotane. Staining of the resected tumor demonstrated patchy positivity for PTH (Fig. 3).

## Discussion

Possible etiologies of hypercalcemia in a patient with ACC on mitotane therapy are varied. Increased renal calcium reabsorption secondary to volume depletion may be prevalent in cancer patients with poor oral intake and in those experiencing gastrointestinal side effects from mitotane [4]. Similarly, the adrenolytic effects of mitotane may also precipitate hypercalcemia secondary to adrenal insufficiency [4]. Malignant hypercalcemia secondary to skeletal metastases and paraneoplastic PTHrp production are also more common reasons for hypercalcemia in patients with ACC [5].

While malignant hypercalcemia is more frequently associated with PTHrp production, rarely it may result from ectopic PTH secretion. A number of PTH-producing tumors have been described in the literature, including PTH-producing myeloblastic leukemia cells, hepatocellular carcinoma, small cell lung cancer, ovarian carcinoma, pancreatic neuroendocrine tumors, papillary thyroid cancer, bronchogenic lung cancer, and pheochromocytomas [6]. Given its rarity, the mechanism behind ectopic PTH production is not well described. In one study in a patient with ovarian carcinoma and hypercalcemia, molecular analysis identified alterations in the regulatory region of the PTH gene, suggesting that dysregulation of PTH gene expression may serve as an underlying mechanism of ectopic PTH production [7]. Other studies have suggested the possibility of PTH gene locus rearrangements or hypomethylation of the PTH promoter [8-10].

The relative contribution of the enlarged parathyroid gland to the patient's hypercalcemia cannot be determined. However, the identification of 2 pathologically normal parathyroid glands alongside a single markedly enlarged gland argues against 4-gland hyperplasia and secondary hyperparathyroidism as the etiology. Given that ectopic PTH production from the ACC would be expected to suppress normal parathyroid tissue, the finding of a single gland enlarged to 10 times normal size is particularly notable and led us to hypothesize that this represented a concomitant primary hyperparathyroidism from a parathyroid adenoma.

In patients who have both hypercalcemia secondary to primary hyperparathyroidism and ACC, genetic syndromes should be considered. While ACC is more commonly sporadic, it is associated with a number of genetic syndromes (Li-Fraumeni, Lynch, Beckwith-Wiedemann, familial adenomatous polyposis, and MEN1); all patients with ACC should be offered hereditary genetic testing [11-13]. Notably, in MEN1, primary hyperparathyroidism is the presenting disease in nearly all cases, and ACC has an incidence of nearly 7% [14, 15].

This novel case of simultaneous steroid and PTH-producing recurrent ACC with parathyroid adenoma included biochemical cure of hyperparathyroidism which occurred only following HIPEC and cytoreduction of recurrent ACC, with subsequent tumor staining demonstrating PTH expression. While it has been estimated that nearly 70% of ACCs are functional, they have rarely been shown to secrete nonadrenal hormones. However, phenotypic shifts in hormone secretion have been reported in recurrent endocrine tumors, including silent pituitary adenomas transitioning to functional tumors [16, 17]. The shift in secretory phenotype observed in this patient, with the primary ACC secreting estradiol and recurrent disease secreting both estradiol and PTH, represents a rarely documented phenomenon in adrenocortical carcinoma. Barzon et al described a similar case in which the ACC secretory profile evolved from primary aldosterone excess to recurrent disease with normalized aldosterone and new steroid hypersecretion, demonstrating that steroidogenic activity can change during tumor progression [18]. This phenomenon is more extensively characterized in pancreatic neuroendocrine tumors (PNETs). In a case-series, de Mestier et al identified metachronous hormonal syndromes in 3.4% of 435 patients with PNETs, associated with rising Ki-67 and worse survival [19]. Crona et al similarly documented changes in hormonal secretion during the disease course in 4% of 323 patients with PNETs, again correlating with more advanced and progressive disease [20]. The underlying mechanism, whether clonal evolution, epigenetic reprogramming, or selection of a pluripotent progenitor cell, remains unknown. In addition, as indicated in Fig. 3B, unequivocal, multifocal cytoplasmic PTH staining was identified in the adrenal cortical carcinoma tissue, which, in light of the clinical and metabolic profile, was diagnostic of tumoral production of PTH by the patient's ACC. This patient did have elevated estradiol levels during follow up. We suspect this was likely secondary to the mitotane effect of elevating sex hormone-binding globulin (SHBG) levels, an increased testosterone substrate for conversion to estradiol via 5-alpha reductase inhibition, and a possible compensatory increase in luteinizing hormone (LH) levels.

Similar to this case, there is one other documented case of ectopic PTH secretion in a male presenting with gynecomastia and an estradiol-secreting oncocytic ACC which was resected following both negative parathyroid imaging and neck exploration [3]. In this case, paraneoplastic hyperparathyroidism caused persistent hypercalcemia despite resection of a presumed functional parathyroid adenoma. Diagnosis was confounded due to a true coexisting parathyroid adenoma masking ectopic PTH secretion and a missing parathyroid intraoperatively. However, biochemical cure of hyperparathyroidism was achieved once the recurrent ACC was resected. This highlights the importance of maintaining paraneoplastic PTH production in the differential for persistent hyperparathyroidism in a patient with a history of metastatic cancer.

## Learning points

- ACC should be recognized as a potential source of ectopic, nonadrenal hormone production, including PTH.
- In a patient with metastatic cancer, PTH production in addition to PTHrp should be considered as a potential paraneoplastic syndrome.

## Data Availability

Data sharing is not applicable to this article as no datasets were generated or analyzed during the current study.

## Abbreviations

ACC: adrenocortical carcinoma
HIPEC: heated intraperitoneal chemotherapy
MEN1: multiple endocrine neoplasia type 1
PNET: pancreatic neuroendocrine tumor
PTH: parathyroid hormone
PTHrp: parathyroid hormone–related peptide
PTX: parathyroidectomy
